# Phosphate oxygen isotopes in lake sediments: stability and application for assessing palaeo nutrient dynamics

**DOI:** 10.1007/s10533-026-01324-0

**Published:** 2026-04-07

**Authors:** Christopher Bengt, Savannah Worne, Peter Wynn, Ana Prohaska, Tim Sexton, Andrew C. Smith

**Affiliations:** 1 British Geological Survey, Nottingham, NG12 5GG UK; 2 Lancaster Environment Centre, Lancaster University, Lancaster, LA1 4YQ UK; 3 Loughborough University, Loughborough, LE11 3TU UK; 4 Globe Institute, University of Copenhagen, 1350 Copenhagen, Denmark; 5 Leicestershire and Rutland Wildlife Trust, Leicester, LE3 5DE UK

**Keywords:** Biogeochemistry, Palaeo reconstruction, Nutrient dynamics, Phosphorus, Stable isotopes, Lake sediments

## Abstract

The oxygen isotope composition of phosphate (δ^18^O-PO_4_) has been successfully used to study modern biological cycling of phosphorus (P) from the upper few centimetres of lake sediments. However, there is a lack of knowledge regarding the stability and preservation of δ^18^O-PO_4_ over longer time scales in deeper lake sediments. Three sediment cores were collected from a nutrient-rich lagoon at Rutland Water Nature Reserve to explore P dynamics under controlled conditions, including a baseline (untreated) core and cores stored with oxygen-enriched water at 4 to 7 °C for six months. Results of the baseline core suggests that P in the sediment has undergone biological turnover. Additionally, results of the two treated cores using δ^18^O-PO_4_ from HCl extractable inorganic P pool remained stable, even under altered water oxygen isotope conditions. These finding offer proof of concept for using δ^18^O-PO_4_ as a tracer of past nutrient inputs and cycling with a range of potential applications in the area of past ecosystem reconstruction.

## Introduction

Phosphorus (P) is a vital element for terrestrial and aquatic life (Smith 1984; Tamburini et al. 2018). Phosphorus is the co-limiting, with nitrogen, nutrient for productivity in terrestrial and aquatic ecosystems (Ruttenberg 2003; Gross et al. 2015; Du et al. 2020). In freshwater lake systems P sources originate from both internal and external to the lake, playing a crucial role in lake system productivity. External loading introduces P from the catchment, including anthropogenic sources, such as sewage effluent, agricultural runoff and industry, and natural sources, such as soil erosion, rainfall and ash deposition (Shoji and Takahashi 2002; Withers and Jarvie 2008; Ji et al. 2017). Meanwhile, internal loading occurs when P stored in lake sediments is released and recycled following changes in pH, temperature, redox potential and other biological processes which trigger the mobilisation of sediment-bound P into the water column (Søndergaard et al. 2001; Ribeiro et al. 2014; Paytan et al. 2017). Understanding the impact of these different sources of P within lakes is critical for effective lake management and restoration. Sedimentary records of P have the potential to elucidate how sources of P and the wider lake P cycle have changed over time. In lake sediments P primarily occurs as (i) iron-bound P (redox sensitive), (ii) aluminium-bound P, (iii) calcium-bound P (apatite and carbonate-associated), (iv) loosely sorbed exchangeable phosphate (Ruttenberg 1992; Jensen and Thamdrup 1993; Hupfer and Lewandowski 2008). Currently, the most common form of P analysis is to assess its concentration within the sediments. The concentration of P can be done as total P, or through the sequential extraction of the sediment (Psenner 1988; Gourley et al. 1994; Helyar 1998; Adu-Gyamfi and Pfahler 2022). Sequential extraction enables the characterisation of P into fractions or “pools”, providing detailed insights into the forms in which P exists within a sediment core. Studies employing sequential extractions have shed light on processes such as sediment–water P exchange, the role of redox conditions in P release, and the contributions of microbial activity to P cycling (Jensen and Thamdrup 1993).

The use of phosphate oxygen isotope composition (δ^18^O-PO_4_) of P extracted from sediments and waters as a complimentary geochemical proxy for understanding P cycling within lakes is a promising novel method. In terrestrial systems established methods tracking the dynamics and uptake of nutrient availability to plants and soils using δ^18^O-PO_4_ are well studied (Angert et al. 2011; Tamburini et al. 2012; Pfahler et al. 2013). The δ^18^O-PO_4_ also has the potential to provide critical insights into the sources, transformations, and pathways of P within aquatic ecosystems, offering a reliable tracer for identifying processes that influence nutrient dynamics (Davies et al. 2014). The δ^18^O-PO_4_ is particularly useful for examining the interactions between biological, chemical, and physical processes in lake systems (Yuan et al. 2019, 2022). However, the majority of current δ^18^O-PO_4_ studies in aquatic ecosystems focus on modern environments, with water collections (Elsbury et al. 2009) or sediments from the upper few centimetres of the sediment column predominant in the literature (Tao et al. 2022; Jin et al. 2023). While these studies provide valuable insights into contemporary P cycling, they do not consider the potential of δ^18^O-PO_4_ as a proxy for past (100’s to 1000’s of years) P cycling. The main reason for not using δ^18^O-PO_4_ in palaeorecords is our lack of knowledge regarding the stability of δ^18^O-PO_4_ over longer timescales when preserved within lake sediments. Specifically, the extent of post deposition biological cycling within the sediment, which can induce oxygen isotopic exchange between the porewater and the host phosphate molecule. The lack of knowledge has limited the use of δ^18^O-PO_4_ as an approach to modern settings, with no long palaeoenvironmental reconstructions from lakes currently existing. The understanding of past lake P dynamics over longer timescales using δ^18^O-PO_4_, would advance our understanding of ecosystem responses to nutrient inputs of P from various sources and over longer timescales. Additionally, δ^18^O-PO_4_ as a novel proxy has the potential to reveal long-term changes in P availability to plants and in broader ecosystem productivity due to nutrient input derived from natural processes such as volcanic ash fall, or from anthropogenic activities associated with pollution.

We hypothesise that P bound to calcium and carbonates within the lake sediments is stable and well preserved in non-acidic lake settings, thus the δ^18^O-PO_4_ should remain unchanged even under conditions designed to elucidate any P cycling. If this is shown to be correct, δ^18^O-PO_4_ from lake sediments has the potential to act as a reliable and well-preserved tracer for past lake P dynamics. To test the stability of δ^18^O-PO_4_, lake sediment cores were extracted from Rutland Water, UK, sequentially extracted to ascertain P concentrations and purified to silver phosphate for δ^18^O-PO_4_ analysis. Secondary core material was stored in isotopically (δ^18^O) enriched water, to investigate the extent to which (if any) biologically mediated isotope exchange still occurred within these historic sediments. These experiments allow us to assess P preservation within sediment layers and evaluate whether δ^18^O-PO_4_ can serve as a robust proxy for reconstructing past nutrient dynamics in temperate lake systems. Our study highlights a widely applicable new palaeoenvironmental proxy, which may prove critical to understanding past P cycling in lakes and surrounding terrestrial ecosystems, under changing climatic and management conditions.

## Materials and methods

### Site description

**Fig. 1:**
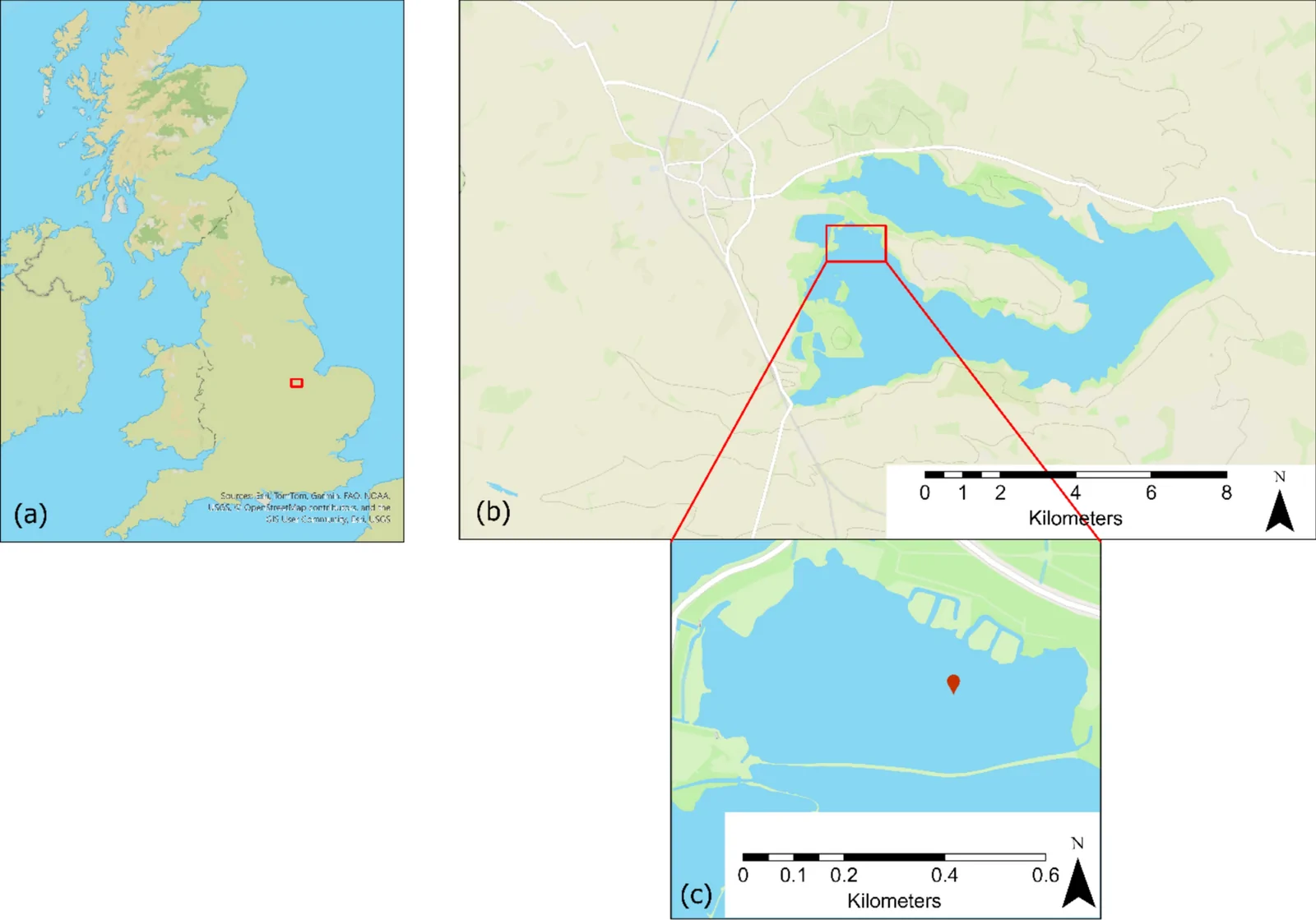
Study site within a national and local context. **a** An overview of the UK and Ireland. **b** An overview of Rutland Water Nature Reserve. **c** LG3 at Rutland Water Nature Reserve. The red pinpoint indicates the coring site. Map produced using software arcGIS Pro (Sources: ESRI, TomTom, Garmin, FAO, NOAA, USGS, © OpenStreetMap contributions, and the GIS User Community, Esri, USGS)

Rutland Water is one of the largest artificial reservoirs in Europe and constructed in the East Midlands, UK, in the 1970s. Combined with the adjacent Rutland Water Nature Reserve (RWNR), composed of 8 shallow water lagoons (< 4 m depth), Rutland Water is a Site of Special Scientific Interest, a European Special Protected Area and internationally recognised as a globally important wetland RAMSAR site. This study focuses on one of the shallow, nutrient-rich water lagoons (max. depth 3.6 m) with an annual mean surface water temperature of 13.3 °C (from Feb 2023 – Feb 2024), and a pH of 8.85 (Feb 2024) in the RWNR – Lagoon 3, hereafter referred to as LG3 (Fig. 1). The main source of water to LG3 is treated effluent from Oakham Water Recycling Centre (WRC; previously sewage treatment works), discharged under permitted consent. With its limited water sourced from the catchment (apart from rainfall and small exchanges with the Rutland Water reservoir and adjacent Lagoon 4 from 2008), LG3 is largely isolated from the larger catchment, providing a unique opportunity to study P nutrient dynamics within a relatively closed aquatic system.

### Sampling methods

In February 2024, three short sediment cores (Core 1, Core 2, Core 3) were collected from LG3 (52°39′41″ N, 000°41′12″ W) using a Kajak gravity corer. After the cores were collected, they were tightly sealed and transported back to the British Geological Survey (BGS) for immediate processing.

**Fig. 2:**
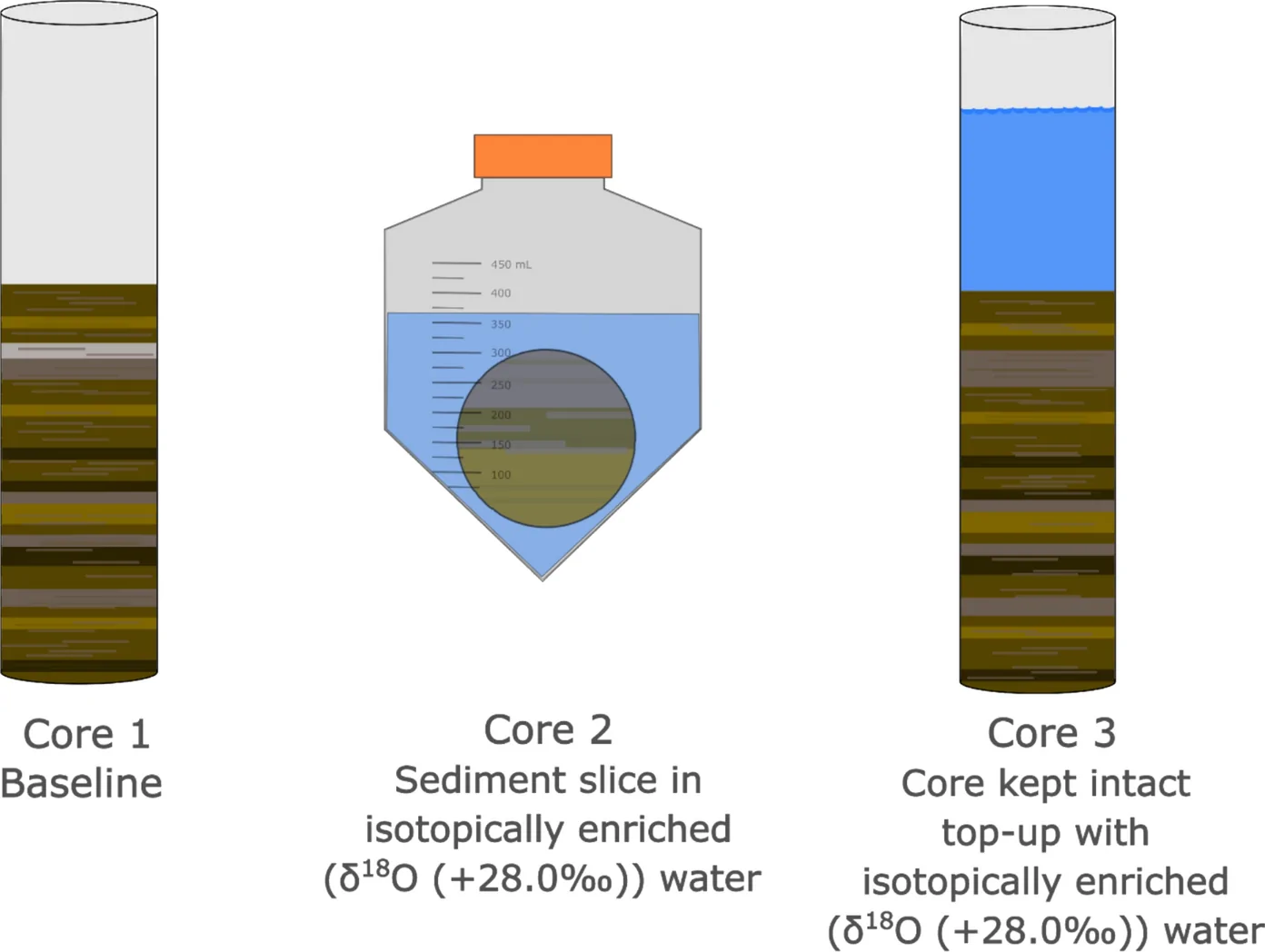
Diagram illustrating experimental set-up for Core 1, 2, and 3

Core 1 was used as a baseline core to assess the natural distribution of P fractions and isotopic composition, so was subsampled and processed immediately (Fig. 2). The core was cut in slices at 1 cm intervals. From each increment, sub-samples were collected for carbon isotope (δ^13^C) and moisture content analysis (with moisture content determined as % weight loss following freeze-drying), pore water extraction and subsequent oxygen isotope in pore water (δ^18^O-H_2_O) analysis (the subsample was immediately frozen and kept in the freezer at − 20 °C until further analysis), the remaining sediment was processed for P concentration via sequential extraction and the extracted inorganic P (P_i_) using hydrochloric acid (HCl) pool processed to silver phosphate (Ag_3_PO_4_) to allow δ^18^O-PO_4_ analysis.

Core 2 was also cut in slices immediately in 1 cm intervals. For each increment subsamples were taken for δ^13^C measurement and moisture content analysis as above. The remaining material was put in 500 mL centrifuge tubes (Coring®) containing 300 mL of isotopically enriched (δ^18^O) water (+ 28.0‰), hereinafter referred to as ^18^O-enriched water, and shaken, maximising the potential for biologically mediated isotope exchange and potential change in the isotopic signature of phosphate (Fig. 2). The samples were then stored at a controlled temperature (4 to 7 °C) for six months to simulate mid- to high latitude basal lake temperatures. After which water samples were extracted for δ^18^O-H_2_O analysis and the remaining sediment processed for sequential P extractions and used the HCl-P_i_ pool to Ag_3_PO_4_ to allow δ^18^O-PO_4_ analysis, as with Core 1.

Core 3 was left intact and upright in the core tube. The surface water in the tube was carefully siphoned off to minimise surface disturbance and replaced slowly with ^18^O-enriched water. The setup was designed to observe how far ^18^O-enriched water would penetrate the core, simulating more natural “in-situ” lake sediment conditions. Again, ^18^O-enriched water was used to enable us to observe if the isotopic signature of oxygen in phosphate changed due to biologically mediated P cycling within the sediment (Fig. 2). The core was then stored at a controlled temperature (4–7 °C), to simulate mid- to high latitude basal lake temperatures, for six months before the core was cut in slices in 1 cm intervals and processed following steps as outlined for Core 1.

### Carbon isotope of sediment cores

Prior to carbon isotope (δ^13^C) analysis, the sediment samples were treated with acid to remove carbonate. Approximately 500 mg of sample was placed in a 50 mL centrifuge tube and immersed with 10 mL of 5% HCl overnight. The samples were then rinsed with MilliQ water until a neutral pH was reached. The samples were then dried overnight at 40 °C to remove any moisture, followed by gently grinding into a fine powder before analysis.

The δ^13^C composition of the sediment samples was analysed using an Elementar vario ISOTOPE cube elemental analyser (EA) coupled to an Elementar isoprime precision isotope ratio mass spectrometer (IRMS), the organic samples were converted into pure carbon dioxide (CO_2_) gas using high-temperature combustion at 950 °C. The stable isotope composition was determined by separating the post-combustion gas into individual molecular species. The data for carbon isotope are reported in delta (δ) notation in per mille (‰) relative to the international reference Vienna Pee Dee Belemnite (VPDB) scale. Normalisation of the carbon isotope ratios were carried out using international standards USGS61 (USGS61 = δ^13^C = -35.05‰ relative to VPDB, U.S. Geological Survey, USA) and USGS62 (USGS62 = δ^13^C = -14–79‰ relative to VPDB, U.S. Geological Survey, USA), and internal standard BROC3 (BROC3 = δ^13^C = -27.6‰). The average external precision (1σ) for the within-run standards and sample repeats was < 0.10 ‰.

### Correlation of sediment cores using carbon isotope and statistical analysis

The three cores collected were of different length and to enable a precise comparison between cores, depth profiles were correlated using δ^13^C values using two tie-points: the top of the core (sediment surface) and the highest δ^13^C depth value were used to align the data against Core 1 as no age model was developed. Correlation of δ^13^C values on the three cores was performed using the QAnalyseries software (Kotov and Pälike 2018). All subsequent results are presented on this scale to allow cross comparison between the cores. The depth of the three cores post-correlation will be reported in “composite depth (cm)”, as the coring—and incubation processes could have a potential to stretch and/or compress the sediment. By using composite depth, this will allow us to align the cores with an accurate depth scale. This composite depth scale is used to compare cores for the rest of this study.

### Sequential phosphorus extraction

**Fig. 3:**
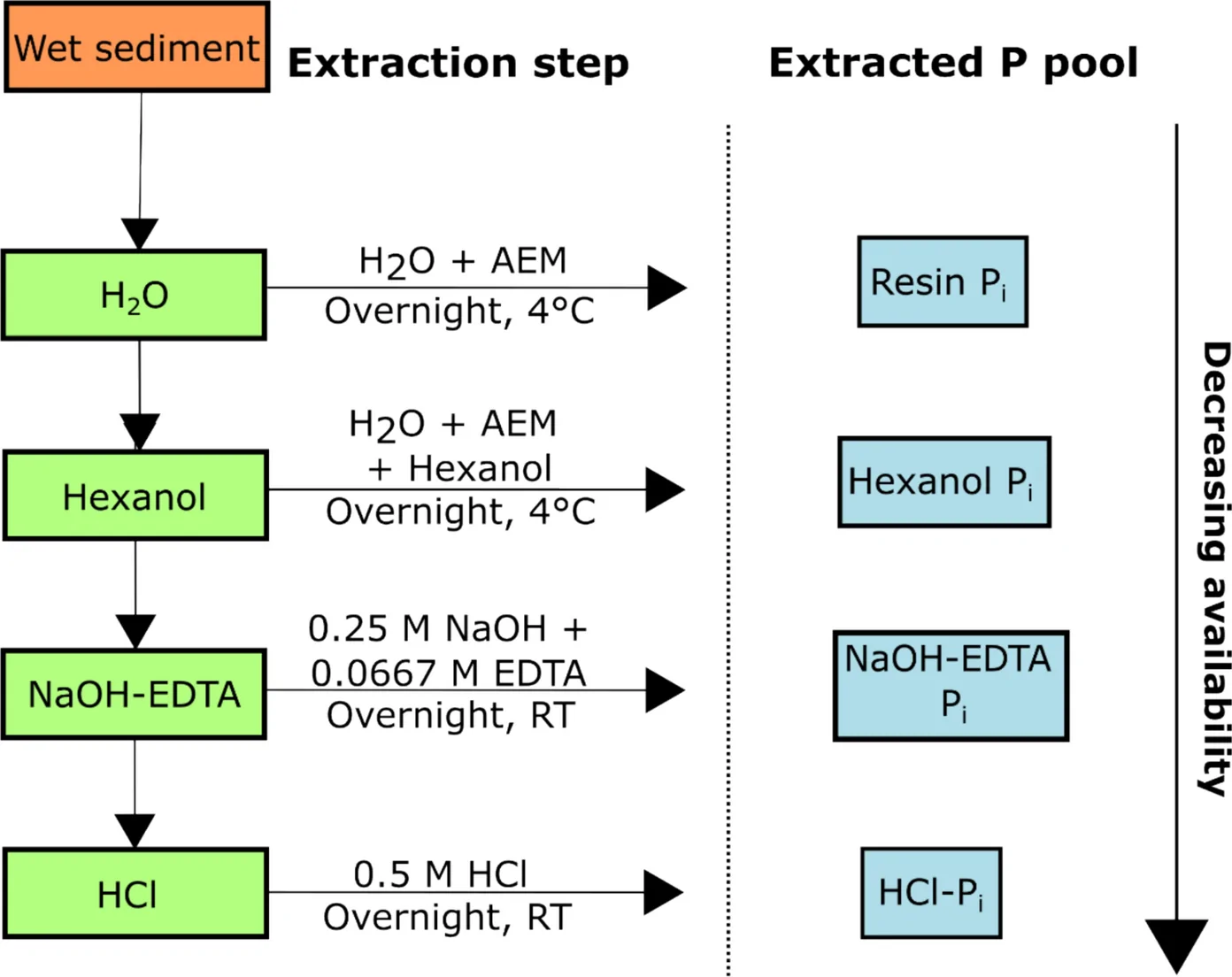
Extraction chart for sequential sediment extraction for selected P pools. P_i_ = inorganic P; H_2_O = filtered water; AEM = anion exchange resin membrane; RT = room temperature. The figure was adapted after Adu-Gyamfi and Pfahler (2022), © IAEA: International Atomic Energy Agency 2022

The sequential P extraction protocol followed methods outlined in (Tamburini et al. 2018) with a few modifications, extracting four different P fractions (Fig. 3). Throughout the sequential extraction, the different P fractions were extracted from the same sediment sample. The four P fractions obtained from the sequential extraction represent different forms of P that can be found in lake sediments. Resin P_i_ represents labile P; this form of P that is loosely bound to the particle surface of sediments and in pore water. The labile P fraction can easily be released from the surface sediments and pore water and be absorbed for bioavailable use (Weiner et al. 2011). Hexanol P_i_ represents microbial P; this form of P that is found within the cell wall of microorganisms including phosphomonoesters and diesters, such as DNA and RNA (Kouno et al. 1995; Adu-Gyamfi and Pfahler 2022). Sodium hydroxide – ethylenediaminetetraacetic acid (NaOH-EDTA) P_i_ represents partly organic P compounds and inorganic P compounds that are bound to metal oxides, such as aluminium and iron (Tamburini et al. 2018). The HCl-P_i_ represents apatite-P and P bound to carbonates, which are obtained from either co-precipitation with endogenic carbonates or from allochthonous lithogenic material. It can dissolve in acidic settings, but the HCl-P_i_ fraction is generally thought to be less bioavailable (Tamburini et al. 2010) and is therefore more likely to have its isotopic signature “permanently” preserved in lake sediments.

The concentration of the extracted P fractions was determined by spectrophotometric analysis (Perkin Elmer Lambda 35, software: UV WinLab) using the molybdenum blue method (Worsfold et al. 2005). The extraction concentrations were converted to µg of P per gram sediment dry weight. The average precision (1σ) of replicate standard measurement (n = 4) was < 0.05 absorbance unites (AU).

### Cryogenic pore water extraction for water isotope analysis

The frozen sediment sub-sample was placed in a cryogenic vacuum distillation apparatus and the air removed as the sample remained frozen, the sediment samples were then gradually heated to a target temperature of 75 °C. Water vapour released from the sediments was transported under vacuum to the cryogenic trap, where it was condensed and collected as liquid. The extracted water was then transferred to a 2 mL airtight glass vial and sealed immediately to prevent evaporation or contamination and stored at 4 °C until isotopic analysis.

Oxygen isotope measurements were conducted using the CO_2_ equilibration method with an Isoprime 100 mass spectrometer coupled with an Aquaprep device. Each water sample (200 µL) was loaded into exetainers (Labco Limited) and placed in a heated sample tray maintained at 40 °C. The exetainers were evacuated to remove atmospheric air, flushed with CO_2_ gas, and left to equilibrate for 12 to 37 h. Following equilibration, the gas from each exetainer was transferred to a cryogenic water trap to remove water vapor. The resulting dry sample gas was then introduced into the dual-inlet mass spectrometer for isotopic analysis. Oxygen isotope data are reported in delta (δ) notation in per mille (‰) relative to the international reference Vienna Standard Mean Ocean Water (VSMOW) scale. Two internal laboratory standards, CA-HI (δ^18^O = − 7.30‰) and CA-LO (δ^18^O = − 39.30‰), were analysed during each run to ensure accuracy. These standards were calibrated against international reference materials VSMOW2 (δ^18^O = 0‰ relative to VSMOW, U.S. Geological Survey, USA), SLAP2 (δ^18^O = − 55.5‰ relative to VSMOW, U.S. Geological Survey, USA), and GISP (δ^18^O = − 33.40‰ relative to VSMOW, U.S. Geological Survey, USA). The average precision (1σ) for the within-run standards was ± 0.05‰.

### Purification and precipitation of silver phosphate

The purification and precipitation protocol of Ag_3_PO_4_ from extracted HCl-P_i_ were slightly modified from the method described by Tamburini et al. (2018). Briefly, the procedure was as follows; ammonium phosphor-molybdate mineral precipitation and dissolution, followed by magnesium ammonium phosphate mineral precipitation and dissolution. These two steps remove any potential organic matter in the sample and concentrates the phosphate within the sample by reducing the volume. These extract steps are followed by a cation removal step, as certain cations have the potential to interfere with the precipitation of Ag_3_PO_4_. Silver ammine (Ag-ammine) solution was added, and samples reacted at 50 °C for 48 h, Ag_3_PO_4_ crystals were filtered and rinsed three times in MilliQ water before being dried and homogenized before analysis.

The analysis of Ag_3_PO_4_ was undertaken by weighing approximately 200 µg of Ag_3_PO_4_ into a high purity silver capsule. The sample was then converted to carbon monoxide (CO) using a thermal conversion elemental analyser (Elementar PYRO cube elemental analyser) at 1450 °C in the presence of carbon sources. The product CO was mixed with a helium carrier gas and was analysed on a Elementar isoprime vision IRMS. The δ^18^O-PO_4_ values were calculated against international Ag_3_PO_4_ standard USGS80 (USGS80 = δ^18^O =  + 13.1‰ relative to VSMOW, U.S. Geological Survey, USA), Ag_3_PO_4_ standards B2207 (B2207 = δ^18^O =  + 21.7‰ relative to VSMOW, Elemental Microanalysis Ltd., England) and internal standard ALFA-2 (ALFA-2 = δ^18^O =  + 14.2‰ relative to VSMOW) was used as a check standard. Samples analysed in duplicate had a typical precision 1σ ≤ 0.5‰. Sample purity was assessed by determining the CO yield compared with the yield of Ag_3_PO_4_ standards (Expected Oxygen % of Ag_3_PO_4_ = 15.3%) and rejecting samples where this differed by > 3%.

### Theoretical equilibrium of phosphate oxygen isotope

Enzymatic activity plays a central role in regulating the isotopic composition of phosphate, as extracellular enzymes such as phosphatases hydrolyse organic P compounds, releasing P_i_ with a distinct isotopic signature (Liang and Blake 2006; Von Sperber et al. 2014). The enzymatic activity links δ^18^O-PO_4_ values to metabolic pathways and biogeochemical cycling, allowing P transformations to be traced and better scrutinised in natural environments (Liang and Blake 2009). Biological activity, such as intracellular metabolism of P_i_ driven by inorganic pyrophosphatase, an enzyme which catalyses the hydrolysis of pyrophosphate to phosphate (Cohn 1958; Blake et al. 2005). This biological activity leads to isotopic exchange between phosphate and water driven by a temperature-dependent equilibrium, influencing the δ^18^O-PO_4_ signature (Davies et al. 2014). To evaluate these processes, theoretical equilibrium δ^18^O-PO_4_ (EQ δ^18^O-PO_4_) values were calculated based on ambient temperature and water isotopic composition using the equation developed by Chang and Blake (2015) and rearranged by Pistocchi et al. (2017).

To calculate EQ δ^18^O-PO_4_, the following equation was employed:

1 $$EQ\delta^{18} O - PO_{4} \; = \; - 0.17 * T + 26.5 + \delta 18O - H_{2} O$$

where: EQδ^18^O-PO_4_ is the equilibrium δ^18^O-PO_4_ value in ‰. T is the ambient temperature in °C (13.3 °C, surface water, for core 1 and 5.5 °C, as an average of 4 to 7 °C, for core 2 and 3). The δ^18^O-H_2_O is the isotopic composition of oxygen in water (‰).

## Results

To understand the extent of biologically mediated phosphate oxygen isotope exchange, data from cores 1, 2 and 3 was compared. The following section details the results of these experiments, highlighting the relative robustness of the HCl-P_i_ pool to enzymatic breakdown within lake sediment cores over the 6-month experimental period.

### Correlation of lake sediment cores

**Fig. 4:**
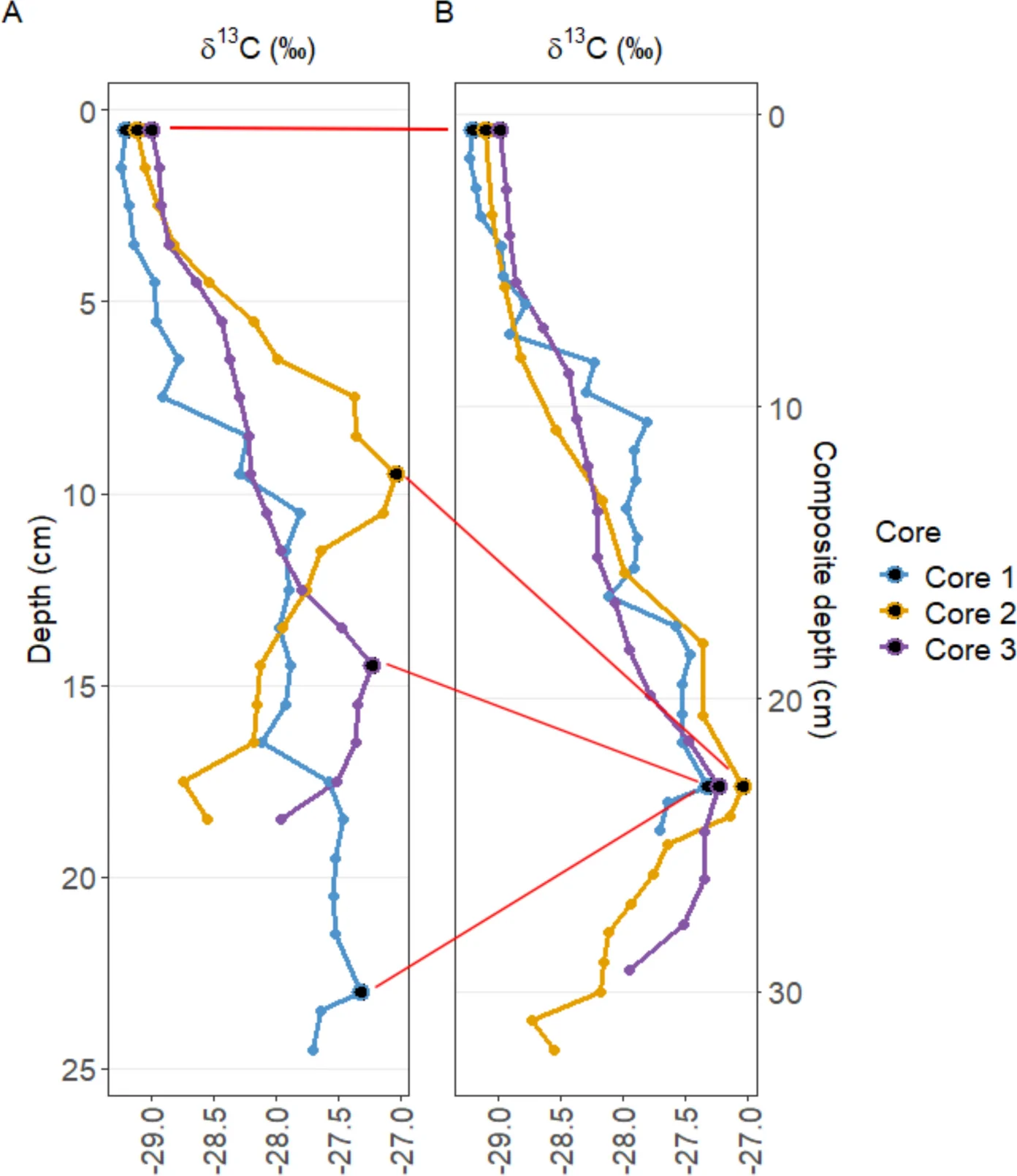
Correlation of δ^13^C: **A** Pre-correlation: δ^13^C of each individual core vs depth, measured before any adjustments or fitting were applied. **B** Post-correlation δ^13^C after fitting to Core 1, which resulted in adjusted values and changes in depth for Core 2 and Core 3. Red lines represent tie points for each core

The isotopic signatures of carbon (C) were analysed for the three lake sediment cores (Fig. 4). δ^13^C values ranged as follows: Core 1 (− 29.23‰ to − 27.32‰), Core 2 (− 29.11‰ to − 27.04‰), and Core 3 (− 28.99‰ to − 27.23‰). These values were used to tie-point to enable comparison between the three cores as described in Sect. Carbon isotope of sediment cores .

### Sequentially extracted phosphorus fractions in lake sediment core

**Fig. 5:**
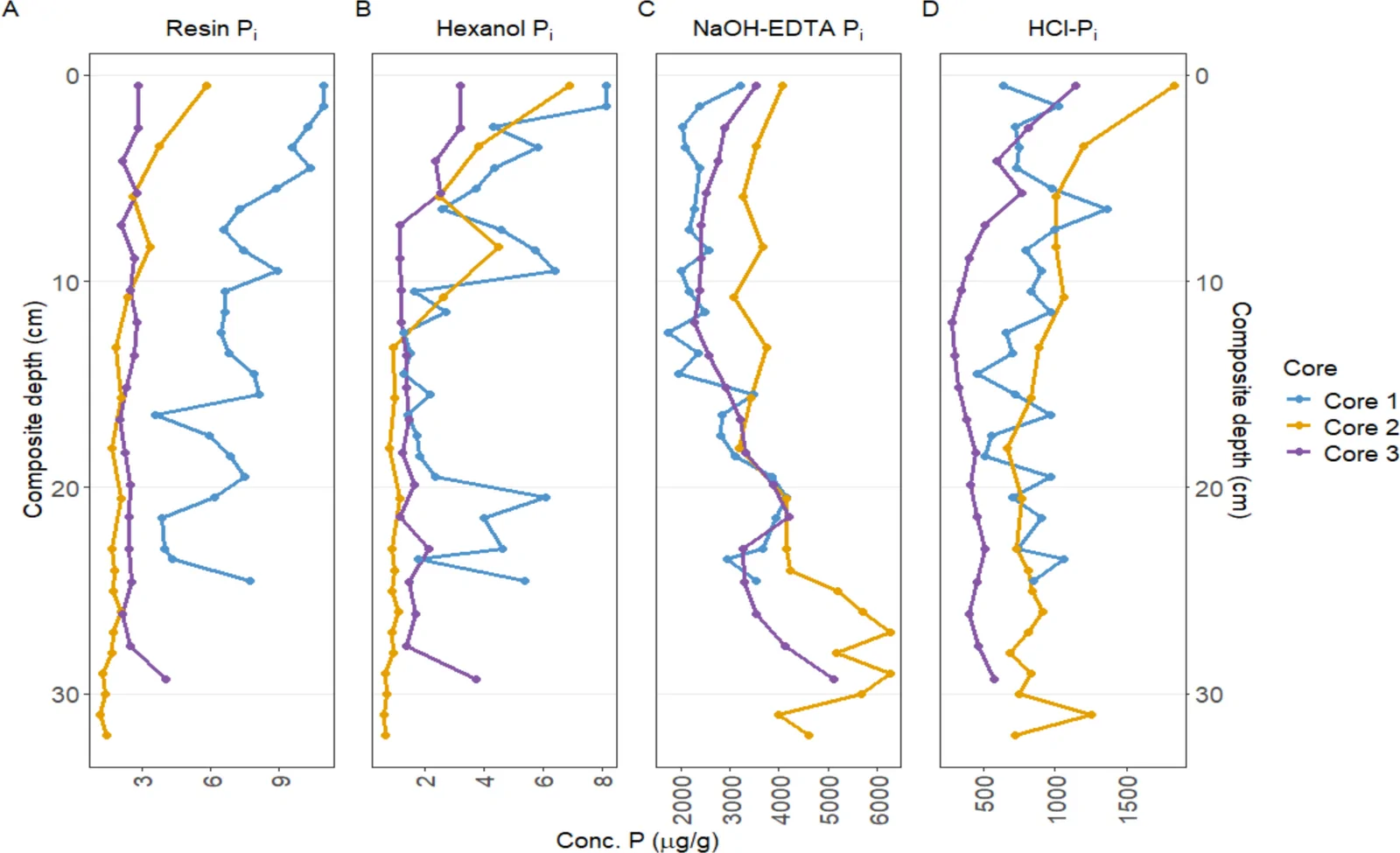
Vertical profiles of sequentially extracted P-fractions. Includes **A** Resin P_i_, **B** Hexanol P_i_, **C** NaOH-EDTA P_i_ and **D** HCl-P_i_ for Cores 1–3

The four extracted P fractions can be grouped into two main categories: bioavailable component (Resin P_i_ and Hexanol P_i_) and inorganic component (NaOH-EDTA P_i_ and HCl-P_i_). The bioavailable component has the lowest concentration of all fractions within each core. Resin P_i_ ranged from 1.20 to 10.92 µg P/g, while Hexanol P_i_ ranged from 0.60 to 8.13 µg P/g. The inorganic componenet represents the dominant P pool in all three cores. NaOH-EDTA P_i_ exhibited concentrations ranging from 1724.88 to 6264.26 µg P/g, while HCl-P_i_ ranged from 281.07 to 1833.86 µg P/g (Fig. 5).

### Oxygen isotope in pore water

**Fig. 6:**
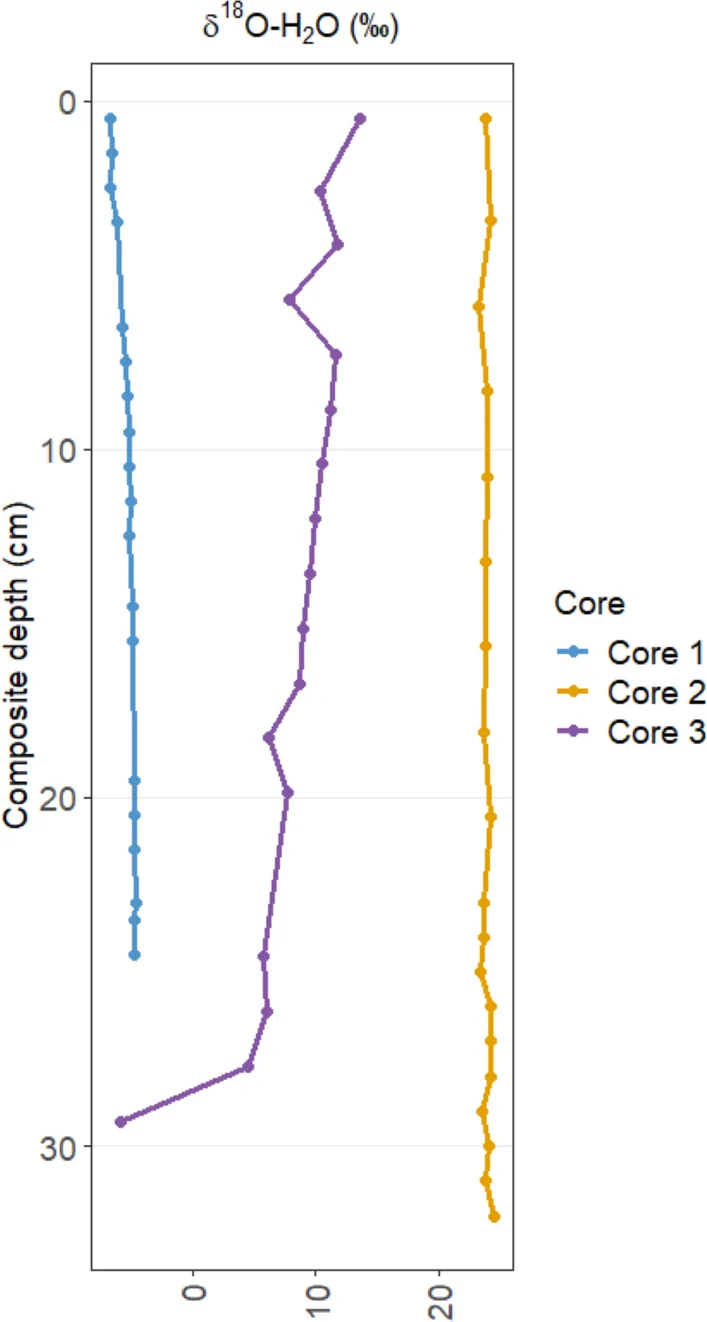
Vertical profiles of δ^18^O-H_2_O from pore water extraction

The δ^18^O-H_2_O values were analysed from the three lake sediment cores (Fig. 6). Core 1 served as the baseline core, with δ^18^O–H_2_O ranging from − 6.70 to − 4.61‰. Core 2 samples were stored refrigerated in ^18^O-enriched water (+ 28.0‰) and, after six months, the δ^18^O-H_2_O ranged from + 23.31‰ to + 24.44‰. Core 3, which was kept intact and had only the surface water replaced with ^18^O-enriched water (+ 28.0‰), showed sediment pore water δ^18^O-H_2_O values ranging from − 5.77‰ at the base to + 13.55‰ at the top after six months.

### Phosphate oxygen isotope

**Fig. 7:**
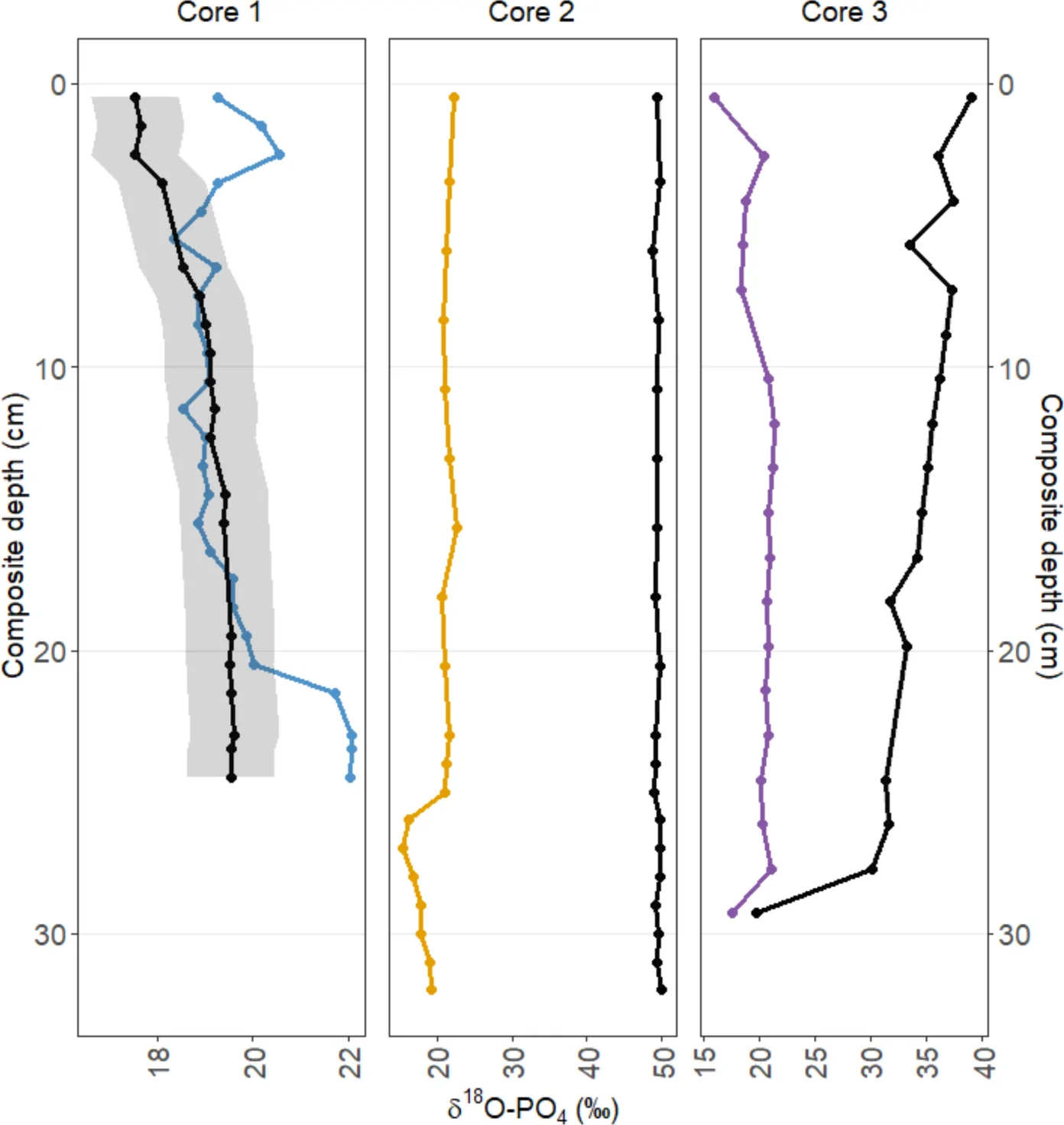
Vertical profiles of δ^18^O-PO_4_ for Core 1, Core 2, and Core 3. The black lines represent the theoretical δ^18^O-PO_4_, calculated using δ^18^O-H_2_O from pore water extraction and for Core 1 the annual mean lake water temperature with ± 5 °C representing seasonal variability (grey area) and Cores 2 and 3 the storage temperature of 4–7 °C

The δ^18^O-PO_4_ values for all three cores are represented in Fig. 7. Each profile also includes the theoretical temperature-dependent equilibrium between oxygen in water and in phosphate (Eq. 1), calculated from measured temperature and δ^18^O-H_2_O. In Core 1, δ^18^O-PO_4_ values range from + 18.36 to + 22.08‰. The theoretical δ^18^O-PO_4_ (black line) remains relatively stable across the profile, with measured δ^18^O-PO_4_ values aligning closely in most of the core, diverging only in the top and bottom layers. For Core 2, δ^18^O-PO_4_ values range from + 15.31 to + 22.71‰. The theoretical δ^18^O-PO_4_ for Core 2 was calculated, assuming temperature dependant equilibration (Eq. 1), to range from + 48.62 to + 50.26‰ based on the enriched water oxygen isotope values added. In Core 3, δ^18^O-PO_4_ values ranged from + 15.92 to + 21.30‰. The theoretical temperature-dependent equilibrium of δ^18^O-PO_4_ for Core 3 was calculated to have a range from + 19.54 to + 39.37‰.

## Discussion

### Core correlation and alignment using carbon isotopes

Collecting lake sediments can cause core stretching when pulling the core up from the lake. For cores, in or case core 3, that have been incubated over time (6 months) the sediment within the core has a chance to recompress over time. To account for this and enable cross correlation / comparison of the cores we used δ^13^C of the sediment to align the three cores. The alignment was performed as no age-model was developed.

### Lake sediment phosphorus and phosphate isotope dynamics in Rutland water

Core 1 served as a baseline core, providing a reference for the natural distribution of P_i_ fractions and δ^18^O-PO_4_ in LG3 sediments. Sequential P_i_ extractions revealed that inorganic component (NaOH-EDTA P_i_ and HCl-P_i_) are the dominating pools within the lake sediment core (99%), while the bioavailable component (Resin P_i_ and Hexanol P_i_) represent a much lower total contribution (1%). The bioavailable component pools unsurprisingly have the lowest concentrations as labile P_i_ is immediately available for biological uptake, and recycling, and is therefore more likely retained within lake waters and entrained within the lakes active biological cycle (Zhou et al. 2001). The NaOH-EDTA P_i_ represents inorganic P compounds that are bound to metal oxides such as iron and aluminium in the sediments (Tu et al. 2019). The second largest fraction, HCl-P_i_, is thought to be more stable over geological periods (Tu et al. 2019). The HCl-P_i_ fraction is commonly bound to calcium and carbonates which are often preserved well in lake sediments (Jin et al. 2006), so the HCl-P_i_ fraction is especially a promising tracer for P dynamics within non-acidic lake settings.

The hypothesis of this study was that δ^18^O-PO_4_ was stable in lake sediments over long time periods and therefore a promising new palaeoclimate and palaeoenvironmental proxy. For this reason, all δ^18^O-PO_4_ extractions were undertaken on the least bioavailable and most geologically stable pool (HCl-P_i_), after sequentially extracting and removing labile microbial and metal bound P pools (Tamburini et al. 2010, 2018). Until now the stability has not been experimentally tested using historical lake sediments, especially those from a highly productive and shallow water setting like LG3 where within sediment P cycling is likely (Søndergaard et al. 2001). The isotopic composition of δ^18^O-PO_4_ in Core 1 reflects the theoretical isotopic equilibrium (Eq. 1), suggesting that P in the lake has undergone significant if not complete turnover. The timing of the turnover cannot, however, be distinguished by Core 1 alone, as it may have occurred either (1) through enzymatic activity whilst in the water column or (2) due to secondary enzymatic breakdown, and isotopic equilibrium within the sediment itself, post deposition.

Interestingly, the first four data points in the top of the core divert from the calculated theoretical isotopic equilibrium (Fig. 7 Core 1). These data points could possibly be due to mixing of the top of the sediment and lake water, entraining phosphate which has not yet fully equilibrated through biological cycling. This hypothesis requires further investigation at this site. The bottom four data points of the core also divert from the calculated theoretical isotopic equilibrium. The divergence is caused by either (1) when the lagoon was created it had a lower trophic status and therefore the phosphate was not fully biologically cycled and the signature is preserved or, (2) a component of the wet chemistry purification has not worked to completion for these few samples. It is not possible with the data we have to conclusively identify the cause of this period of disequilibrium, although a wet chemistry failure seems unlikely as all the samples were processed at the same time using the same procedure and chemicals. However, studies at LG3 continue with the aim of conclusively resolving this. The disequilibrium raises the critical question of how stable δ^18^O-PO_4_ is within organic rich lake sediments and therefore, to what extent can sedimentary δ^18^O-PO_4_ be used as a palaeoclimate proxy for the lake water P cycle? To test whether δ^18^O-PO_4_ can be used as a proxy for palaeoreconstruction, cores 2 and 3 were subjected to long term (6 month) storage experiments with isotopically enriched water, to trace any isotope exchange that occurred due to “in core” enzymatic exchange.

### Determining the stability of phosphate oxygen isotope composition in lake sediments

The experimental design for cores 2 and 3 was set up to determine the extent of preservation of the original lake sediment δ^18^O-PO_4_ within sediment layers when exposed to ^18^O enriched water and simultaneously explore if any large-scale changes in P pool concentrations were observed due to the removal of the core from the lake bottom. The sequential extraction of cores 2 and 3 after six months of incubation, revealed that the inorganic component (NaOH-EDTA P_i_ and HCl-P_i_) remains the dominating fractions, 82 and 17% respectively. The concentrations of the bioavailable component are still significantly lower (< 1%) than the inorganic component. Over the six-month incubation period, a decrease in the concentrations of both bioavailable pools was observed (from average down core values of core 1 for resin P_i_ 7.34 µg P/ g to 2.20 µg P/ g of core 2 and of core 1 for hexanol P_i_ 3.75 µg P/ g to 1.71 µg P/ g of core 2). This decrease in the concentrations most probably indicates a small turnover and re-mineralisation of these minor P pools (Faul et al. 2005). Previous studies within the soil science community have demonstrated that the bioavailable component can be exchanged within months or even minutes and mineralisation can also occur rapidly (Helfenstein et al. 2020). Whilst slight, this change in P pool composition is important to note, in case of biological cycling and re-mineralisation of the resin P_i_ and hexanol P_i_ pools has any impact on mineral (HCl-P_i_) isotope values.

The isotopic composition of the pore waters (δ^18^O-H_2_O) in cores 2 and 3 reflect the isotopically enriched water added to the sliced sub-samples, demonstrating the expected increase in δ^18^O-H_2_O compared to the original pore water values. Average values in Core 2 (+ 23.90‰) show a mixed δ^18^O-H_2_O value, a combination of the original sediment water isotope value and the added ^18^O-enriched water (+ 28.0‰). The new isotopic composition of Core 2 samples was stable due to complete mixing, achieved by artificially shaking each sliced sample with enriched water. In Core 3, which only had isotopically enriched water added to the surface of the undisturbed sediment, the δ^18^O-H_2_O (

Figure 6, purple line) indicates that ^18^O-enriched water diffused through the intact sediment core and mixed with almost all of the pore water in the sediment (1 to 27 cm depth). The finding indicates the potential for significant water diffusion within lake sediments, especially cores stored for long periods of time. The diffusion within lake sediments is possible something that should be considered by researchers storing lake sediment cores for future work, not only for isotope analysis, but also for other kinds of work such as ancient DNA analysis. The isotopic value at the bottommost of the core remained unchanged, reflecting the original lake water isotope value (-5.29‰), likely due to the presence of a barrier (bung) at the base of the core and resultant pressure that prevented further diffusion.

The significant δ^18^O-H_2_O shift in cores 2 and 3 from the baseline (Core 1), forced through the addition of ^18^O-enriched water, allows us to observe any change in δ^18^O-PO_4_ as a result of large scale, enzyme driven phosphate breakdown (Eq. 1). The theoretical temperature-dependent equilibrium of these cores was shifted significantly by + 29.19‰ (Core 2) and + 13.46‰ (Core 3) from the theoretical temperature-dependent equilibrium of Core 1. However, even after 6 months, the δ^18^O-PO_4_ (HCl-P_i_ fraction) of both core 2 and 3 remained unaffected, retaining similar isotopic ranges as Core1 (+ 19.93‰ and + 19.96‰ respectively), being out of equilibrium with the new surrounding pore water. This disequilibrium from the theoretical temperature-dependent equilibrium was the case even when the sediment cores have been under conditions designed to maximise sediment/water interaction (Core 2) as intracellular reactions between oxygen in P_i_ and oxygen in in surrounding water can exchange rapidly, in order of minutes (Davies et al. 2014). Whilst these experiments were reasonably short term, 6 months, on a longer timescale, years to millennia, we would have expected to see some minor deviation in δ^18^O-PO_4_ of both cores 2 and 3 if biologically mediated exchange was occurring at a relevant rate. These findings are therefore encouraging and suggest that the δ^18^O-PO_4_ (HCl-P_i_ fraction) values are not altered significantly by post-depositional biological turnover in temperate lake sediments in colder high latitude conditions (4 to 7 °C).

The stability of δ^18^O-PO_4_ in this study aligns with previous oxygen isotope studies on marine sediments (Jaisi and Blake 2010; Blake et al. 2010; Joshi et al. 2015), which have shown that δ^18^O-PO_4_ values in sediments are often resistant to isotopic exchange once phosphate has been incorporated into metal ions and carbonates. Additionally, (Helfenstein et al. 2020) demonstrated that phosphate in soils associated within the inorganic component exhibits varying turnover rates, with the NaOH-EDTA pool exchange between 1 h and 3 months while the HCl-P_i_ pool persisting for years to millennia. The results from cores 2 and 3 reinforce this distinction, as the δ^18^O-PO_4_ values remained completely unaltered despite experimental conditions designed to enhance P turnover (core 2) and highlight even small levels of enzymatic P cycling. These results indicate the long-term stability of sedimentary δ^18^O-PO_4_ and the potential to use lake sediment δ^18^O-PO_4_ as an exciting new tracer for reconstructing past P cycling in lake systems.

## Conclusion

This study undertook laboratory experiments to investigate P cycling and isotopic stability in lake sediments, providing valuable insights into the preservation of δ^18^O-PO_4_ over time (6 months). By analysing sediment cores from a shallow water lagoon in Rutland Water Nature Reserve, exposed to ^18^O-enriched conditions, we assessed the potential for δ^18^O-PO_4_ to serve as a proxy for palaeo P dynamics in temperate freshwater environments. Our findings demonstrate that δ^18^O-PO_4_ associated with HCl-P_i_ remains stable over extended periods in lake sediments, as with previous studies in marine and soil environments; even under ^18^O-enriched conditions designed to enhance and highlight P cycling. Further research should explore the stability of δ^18^O-PO_4_ of the NaOH-EDTA P_i_ pool and the HCl-P_i_ pool under varying environmental conditions, including warmer temperatures and different redox regimes, to determine its applicability across a broader range of latitudinal and chemical lake settings. Additionally, exploring whether different concentrations of P within the sediments has an influence in preserving the isotopic signature of the HCl-P_i_ pool. Overall, this study provides a critical step towards understanding the long-term preservation of δ^18^O-PO_4_ in freshwater sediments, laying the foundation for future applications of the δ^18^O-PO_4_ proxy in palaeoclimatic and palaeoenvironmental reconstruction, eutrophication monitoring, and nutrient management strategies.

## Data Availability

Data is available through the National Geoscience Data Centre (NGDC) hosted by the British Geological Survey, https://doi.org/10.5285/62867651-fde7-4f90-86db-5a23f55c906a (Bengt 2026).
